# Phase separation of planetary ices explains nondipolar magnetic fields of Uranus and Neptune

**DOI:** 10.1073/pnas.2403981121

**Published:** 2024-11-25

**Authors:** Burkhard Militzer

**Affiliations:** ^a^Department of Earth and Planetary Science, University of California, Berkeley, CA 94720; ^b^Department of Astronomy, University of California, Berkeley, CA 94720

**Keywords:** giant planets, solar system, ab initio simulations

## Abstract

The Voyager spacecraft discovered that the ice giants Uranus and Neptune have nondipolar magnetic fields, defying expectations that a thick interior layer of planetary ices would generate strong dipolar fields. Stanley and Bloxham showed that nondipolar fields emerge if the magnetic field is only generated in a thin outer layer. However, the origin and composition of this dynamo active layer has so far remained elusive. Here, we show with ab initio computer simulations that a mixture of H_2_O, CH_4_, and NH_3_ will phase separate under the pressure–temperature condition in the interiors of Uranus and Neptune, forming a H_2_O-dominated fluid in the upper mantle and a CH_4_-NH_3_ mixture below. We further demonstrate that with increasing pressure, the CH_4_-NH_3_ mixture becomes increasingly hydrogen depleted as it assumes the state of a polymeric C-N-H fluid. Since the amount of hydrogen loss increases with pressure, we propose that the C-N-H fluid forms a stably stratified layer. The magnetic fields are primarily generated in an upper layer that is H_2_O-rich, homogeneous, convective, and electrically conducting. Under these assumptions, we construct ensembles of models for the interiors of Uranus and Neptune with the Concentric MacLaurin Spheroid method. We demonstrate that the phase separation of the solar-type H_2_O-CH_4_-NH_3_ mixture leads to models that match the observed gravity field and to layer thicknesses that are compatible with magnetic field measurements.

## Significance

The Voyager spacecraft measured that Uranus and Neptune have nondipolar magnetic fields while strong dipole fields had been expected. Stanley and Bloxham thus proposed that the magnetic fields be generated only in a thin outer layer. Here, we predict what the materials in the interior layers are and why the lower layer is dynamo inactive. We demonstrate with ab initio simulations that planetary ices phase separate at high pressure into an upper, water-rich and a lower, hydrocarbon-dominated layer. The upper layer is convective and dynamo active while the lower layer is stably stratified. A signature of the stratification can be detected in normal modes, which lends support to placing a Doppler imager on a future Uranus mission.

To characterize the structure and evolution of giant planets, it is essential to know what types of layers exist in their interiors. While orbiting spacecrafts like Juno measured the gravity field with exquisite precision, it is still possible to match these measurements with different types of interior structure models (1). Information about interior layers and their properties may be inferred from other types of data. Fuller et al. (2) for example showed that a stably stratified layer must exist in Saturn’s interior to explain the splitting of normal modes that was detected with ring seismological observations. To explain an excess in Saturn’s luminosity, Stevenson and Salpeter (3) predicted that hydrogen and helium become immiscible in the planet’s interior, which introduces a helium rain layer and provides an energy source that explains the luminosity excess. It took many years before these predictions were confirmed with ab initio computer simulations (4, 5) and laboratory experiments (6).

Few constraints exist for the interior structure of Uranus and Neptune because these planets have so far only been visited by one spacecraft, Voyager 2. The interpretation of these gravity measurements has not been unique (7) but it is generally assumed that a large part of their interior is composed of planetary ices, H_2_O, CH_4_, and NH_3_ (8). The most important constraint about their interior structure might come from the properties of their magnetic fields. The Voyager 2 spacecraft determined that both planets have nondipolar magnetic fields, which was rather unexpected. Following up on Ruzmaikin and Starchenko (9) and Hubbard et al. (10), Stanley and Bloxham (11, 12) performed numerical dynamo simulations and demonstrated that nondipolar magnetic fields emerge if they are generated primarily in a thin outer layer, between fractional radii of 2/3 and 1 for example. The authors reproduced the observed field morphologies best if they assumed a stably stratified, electrically conducting, liquid layer resides below the dynamo active layer. They also considered the case in which the lower layer is a nonconvective solid that is electrically conducting. A rigidity of a solid would, however, anchor more strongly the magnetic field lines, which emerge from the upper layer, and this anchoring effect would ultimately cause the simulations to reproduce the observed fields less accurately. The concept of a stably stratified liquid lower layer was therefore favored.

The concept of a solid inner layer was nevertheless favored by the authors of refs. 13–15 who argued that superionic H_2_O might behave like a nonconvecting solid in the interiors of Uranus and Neptune. This concept was disputed by Matusalem et al. (16) who demonstrated with ab initio simulations that superionic water has a very low viscosity and that plastic flow occurs easily under the conditions in ice giant interiors.

Furthermore, the formation of diamond from CH_4_ in ice giant interiors has been explored with theoretical and experimental techniques (17–19). As an alternative explanation for the nondipolar fields, Soderlund et al. (20) performed magneto-hydrodynamic simulations of Uranus’s and Neptune’s magnetic fields assuming thick and thin shell dynamos. They suggested that the nondipolar magnetic fields are the results of turbulent convection that is driven by thermal buoyancy. Various dynamo types are discussed in ref. 21.

The structure and evolution of Uranus and Neptune has been studied with a variety of methods and assumptions as the review by Helled et al. (22) illustrates. Nettelmann et al. (23) matched the available gravity data with interior models that have three layers, each being homogeneous and convective. The outer two layers are mixtures of hydrogen, helium, and water but they differ in composition. The third layer is a rocky core. Helled and Bodenheimer (24) constructed core-accretion models for Uranus and Neptune and studied conditions that led to the observed masses and solid-to-gas ratios. Bailey and Stevenson (25) interpreted the difference in heat flux between Uranus and Neptune as a sign of dissimilar degrees of water–hydrogen mixing in the planets’ outer envelopes. Stixrude et al. (15) studied the thermal evolution of Uranus’s interior and suggested that the absence of a strong heat flux could be the result of a growing core that is made of superionic water. Movshovitz and Fortney (7) constructed ensembles of interior models for Uranus and Neptune with agnostic pressure-density relationships and then constrained the moments of inertia and discussed measurements of a future low-periapse orbiter. Recently Neuenschwander et al. (26) studied the possible relationships of pressure, density, temperature, and composition by introducing convective and nonconvective layers into Uranus’s interior and compared models that include a water-rich layer with models that do not.

In this paper, we favor the hypothesis of two liquid layers and provide a material-based explanation. With ab initio simulations, we show that a homogeneous fluid mixture of planetary ices spontaneously phase separates into a water-rich phase and a C-N-H phase. Both are good electrical conductors. We demonstrate furthermore that with increasing pressure, more and more hydrogen is released from the C-N-H fluid as it attains a polymeric structure where many C and N nuclei are bonded to each other. This introduces a chemical gradient into the lower layer, which stabilizes it against convection. We thus conclude that the magnetic field is generated only in the upper, water-rich layer. With the Concentric MacLaurin Spheroid method (27, 28), we then construct models for the interiors of Uranus and Neptune that match the observed gravity field and show that the layer boundary at a fractional radius of 2/3 is broadly compatible with the assumed protosolar mixture of planetary ices.

## Results from Ab Initio Simulations

**Fig. 1.**
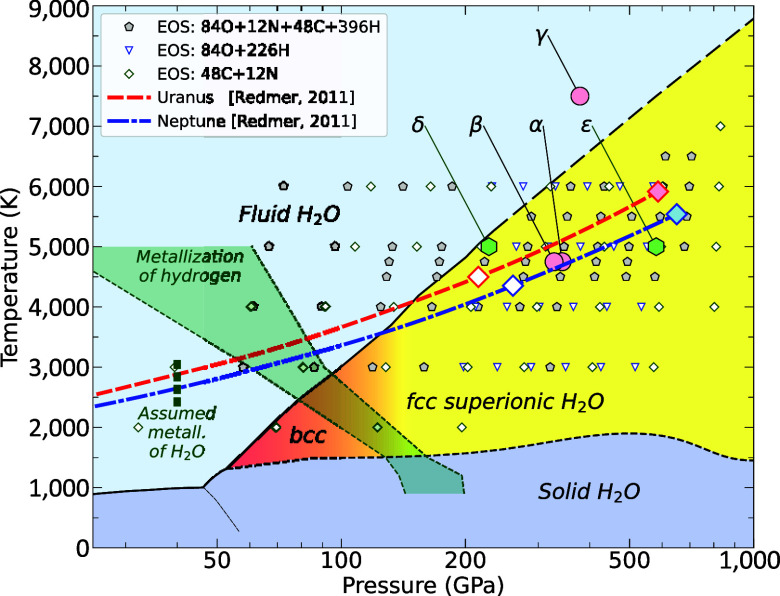
Phase diagram of H_2_O based on refs. 29–31. The small symbols indicate conditions of ab initio equation of state calculations of different materials that we conducted. The Greek labels specify conditions of simulations that are discussed in Figs. 2–4. The thick red and blue-dashed lines represent the isentropes of Uranus and Neptune from Redmer et al. (32). The open and filled diamonds respectively show conditions at the *Upper* and *Lower* ends of the C-N-H layers of the interior models in Fig. 5. The vertical dashed line marks the pressure of 40 GPa at which Stanley and Bloxham assumed pure H_2_O to become sufficiently conducting, 20 S/cm, to become dynamo active. The green shaded region marks conditions from ref. 33 where hydrogen is predicted to attain a reflectivity of 0.3 (*Left* boundary) and a conductivity of 2,000 S/cm (*Right* boundary).

**Fig. 2.**
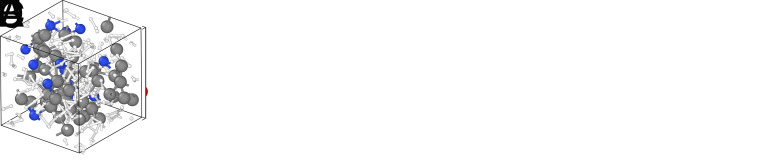
Panels (*A* and *B*) show the same snapshot from our simulation, α, of 12 × [7H_2_O + 4CH_4_+ NH_3_] atoms at 343 GPa and 4,750 K. The oxygen, hydrogen, carbon, and nitrogen atoms are depicted in red, white, gray, and blue colors. Panel (*B*) shows only the carbon and nitrogen atoms that are concentrated in the cell center to emphasize that they phase separate from the oxygen-rich fluid. This phase separation can be seen in panels (*C* and *D*), which shows a snapshot from our hydrogen-depleted simulation, β, of composition 12 × [7H_2_O + 4CH_4_+ NH_3_] − 164H atoms at similar conditions of 328 GPa and 4,750 K. The carbon and nitrogen atoms are concentrated on the *Left* side. Panel (*E*) shows our oxygen-free simulation, ϵ, of composition 12 × [4CH_4_+ NH_3_] at 580 GPa and 5,000 K. Here, we added bonds to emphasize that at high pressure, carbon and nitrogen atoms form a dense network of bonds while many hydrogen atoms are released.

In this paper, we assume the planetary ices were delivered to Uranus and Neptune in the ratio 7 × H_2_O, 4 × CH_4_, and 1 × NH_3_ because it approximately represents protosolar ratio of the heavy nuclei (34). We constructed the largest possible supercell with 540 atoms (12 × [7H_2_O + 4CH_4_+ NH_3_]) for which we could afford to conduct ab initio molecular dynamics simulations. Starting from a homogeneously mixed fluid, we performed simulations of a wide range of pressure–temperature conditions shown in Fig. 1 that include the interior of Uranus and Neptune (32). Along their adiabats at 343 GPa and 4,750 K (conditions we labeled α) our ab initio simulations show that the homogeneous fluid spontaneously phase separates into a water-rich fluid and a C-N-H fluid as we illustrate in Fig. 2. We also observe this phase separation in simulations from which we have removed 164 hydrogen atoms (labeled β), which demonstrates that the phase separation is not sensitive to the hydrogen concentration. The simulation results in panels Fig. 2*A*–*D* were obtained with standard ab initio simulations without any machine learning acceleration.

**Fig. 3.**
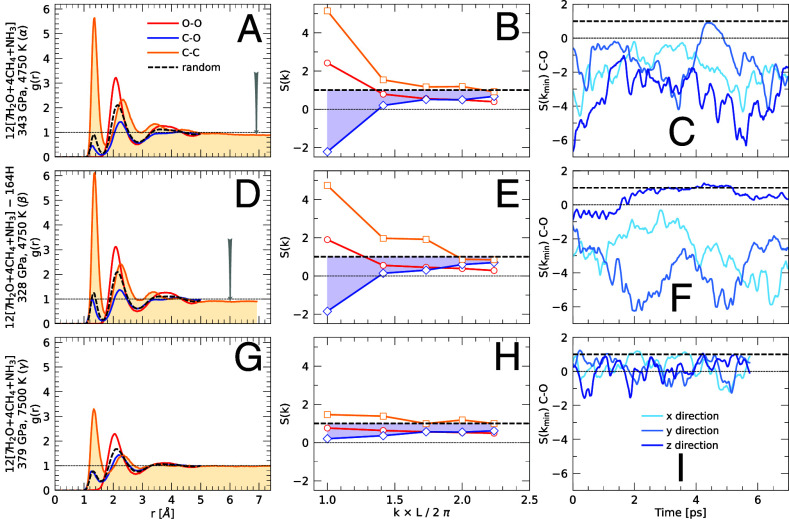
This figure shows pair correlation functions and structure factors. Panels (*A*–*C*) shows results from our simulation, α. The C-C peak at r=1.4Å and the O-O peak at r=2.2 illustrate that carbon and oxygen atoms are preferentially surrounded by atoms of their own type. For r<3, the C-O pair correlation function is consistently lower than a randomized nuclear ensemble (dashed line). We cut off all functions at r=5 except the C-C pair correlations to emphasize that this function drops below 1 for large r (see arrow) to illustrate the trend for the carbon nuclei to separate from the oxygen nuclei. The trend to phase separate is confirmed by the negative value of the C-O structure factor for $k_{\min}=2\pi /L$ in panel (*B*). L is the length of the simulation cell. In panel (*C*), we show how the C-O structure factor for $k_{\min}$ in directions x, y, and z changes as a function of simulation time to emphasize that it is predominantly negative. Panels (*D*–*F*) shows the corresponding results for our hydrogen-depleted simulation β at similar conditions, which also phase separates. Panels (*G*–*I*) show results from the higher-temperature simulation, γ, at 7500 K.

In Fig. 3, we study the short and long-range order in simulations α, β, and for a higher temperature condition label γ. We compute the pair correlation functions $g_{\mathrm{AB}}\left(r\right)$ between different types of nuclei, A and B, as well as its Fourier transform, the structure factor, $S_{\mathrm{AB}}\left(\vec{k}\right)$,

[1] $$\begin{array}{cc} g_{\mathrm{AB}}\left(r\right) & =\frac{V}{4\pi r^{2}N_{A}N_{B}}\sum_{i=1}^{N_{A}}\sum_{j=1}^{N_{B}}\langle \delta (|{\vec{r}}_{i}-{\vec{r}}_{j}|-r)\rangle \;\mathrm{and}\\ S_{\mathrm{AB}}\left(\vec{k}\right) & =\frac{1}{\sqrt{N_{A}N_{B}}}\sum_{i=1}^{N_{A}}\sum_{j=1}^{N_{B}}\langle \exp\left\{-i\vec{k}\left({\vec{r}}_{i}-{\vec{r}}_{j}\right)\right\}\rangle , \end{array}$$

where $N_{A}$ and $N_{B}$ specify the number of particles per type in volume, V. The high C-C and O-O peaks in panel (1*a*) of Fig. 3 show that carbon and oxygen atoms exhibit a strong preference to be surrounded by atoms of their own type. For comparison, we computed the g(r) function for a *randomized nuclear ensemble* where we swapped the types of C, N, and O atoms at random but left their positions unchanged. When one integrates $g_{\mathrm{CC}}\left(r\right)$ up to first minimum at r=1.7 Å, one finds that a carbon atom is on average surrounded by 1.5 other carbon atoms while in the randomized ensemble, there would only be 0.25 carbon atoms with that radius. Conversely, the first and second peak of the $g_{\mathrm{CO}}\left(r\right)$ correlation function in the simulation are much lower than that of the randomized ensemble indicating a tendency for carbon and oxygen atoms to avoid each other on short distances. For large distances, the C-C correlation functions in panels (*A* and *D*) of Fig. 3 drop below 1.0 implying fewer than normal carbon atoms are present at large distances. We observed a similar drop in ref. 35 when a helium bubble formed in hydrogen. At a higher temperature of 7,500 K, one still finds a prominent first peak in the $g_{\mathrm{CC}}\left(r\right)$ function, but for large distances, this function is very close to 1 (see panel *G* of Fig. 3).

We performed a cluster analysis to illustrate how inhomogeneously the heavy nuclei are distributed in the fluid under different conditions. For simplicity, the hydrogen atoms were excluded. We introduce a cutoff distance of 1.8 Å to determine which atoms are bonded to each other. A cluster is a group of atoms that are connected by such bonds. We determined abundance of different clusters by analyzing all configurations in a trajectory. We find that the behavior of the oxygen atoms differs substantially from that of the carbon and nitrogen nuclei. For simulation α, we find that 73% of the oxygen atoms are not bonded to other nuclei while 62% of the carbon and nitrogen nuclei reside in clusters with six atoms or more. This fraction increases to 92% in simulation β that includes fewer hydrogen atoms while the fraction of single oxygen atoms drops to 57% because some oxygen atoms form short-lived bonds with the carbon and nitrogen nuclei. In the oxygen-free simulation δ at 228 GPa, 68% of the carbon and nitrogen nuclei occur in clusters with six atoms or more. In simulation ϵ at a higher pressure of 580 GPa, this fraction increases to 88%. While the values of fractions depend on the cutoff distance, the behavior does not change qualitatively if one varies the cutoff between 1.6 and 2.0 Å.

In Fig. 3, we show that the carbon–oxygen structure factor becomes negative for the smallest $k_{\min}$ vectors ([2π/L,0,0], [0,2π/L,0], [0,0,2π/L]) that fits into our simulation cell of size L. If a pair of carbon and oxygen atoms is separated by large distance like L/2, they make a negative contribution to the $S_{\mathrm{CO}}\left(k\right)$ average in Eq. **1**. If the system has phase separated, there are not enough carbon–oxygen pairs at small distances to make compensating, positive contributions. Therefore, $S(k_{\min})<0$ is an indicator for phase separation. Since X-ray diffraction experiments measure the structure factor, we propose such experiments be conducted on planetary ices at high pressure (36) to verify the phase separation that we have predicted here with ab initio simulations. In the last column of Fig. 3, we show how the $S_{\mathrm{CO}}\left(k\right)$ functions for the three smallest k vectors change as a function of time. All $S_{\mathrm{CO}}\left(k\right)$ vary with time indicating that the shape of carbon–nitrogen bubble in Fig. 2 changes dynamically but one finds that two out of three values are always strongly negative, except for the high-temperature simulation, γ, that appears to be more mixed.

**Fig. 4.**
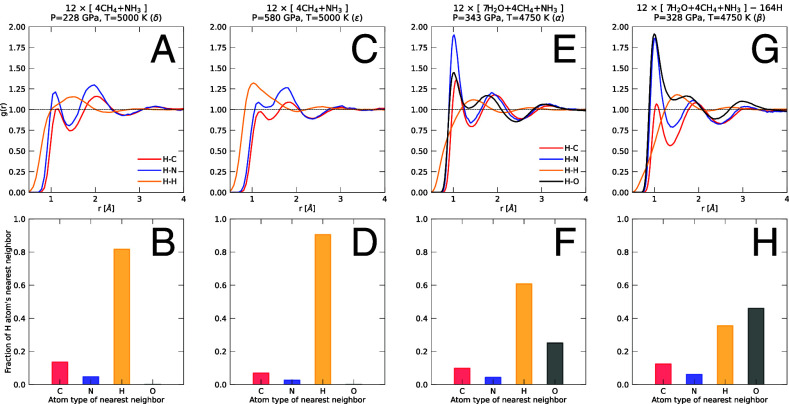
Pairwise the panels (*A, B*), (*C, D*), (*E, F*), and (*G, H*) show results from four simulations at different conditions that are described above the *Upper* panels. The Greek symbols show these conditions in Fig. 1. The *Upper* panels show the pair correlation functions between hydrogen and the other nuclei. As pressure increases fewer H atoms are bonded to C and N nuclei, which manifests itself in decrease in the height of the first peaks of the H-C and H-N functions in panels (*A*) and (*C*). The same panels also show an increase in the height and width of the first peak of the H-H nuclei indicating more H atoms are bonded to each other (see Fig. 2*E*). This trend is confirmed by results in the lower panels where we plot what type of nucleus is the closest neighbor of any given hydrogen atom, averaged over an entire trajectory. The height of the yellow bar shows that many hydrogen atoms are bonded to each and that their fraction increases with pressure. The two panels on the right show results from simulations that contained oxygen as well. The bar charts and the height of the first peaks show that hydrogen atoms have a preference for bonding with oxygen.

**Fig. 5.**
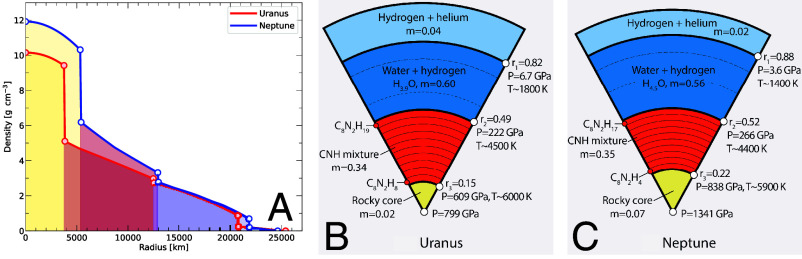
As function of the sphericalized radius, panel (*A*) displays the density profile of the models for the interiors of Uranus and Neptune that we show in panels (*B* and *C*). In these panels, *m* specifies the fractional mass of every layer. The fractional radii and the pressures at the layer interfaces are given on the *Right* side. More details are given in Table 1. The inner and outer dashed lines mark where the transition from a liquid to a superionic state is predicted to occur in pure H_2_O (31) and the pressure of 40 GPa that was assumed to be the outer pressure of the dynamo active layer in ref. 12.

With Figs. 2*E* and 4, we show the C-N-H fluid releases more and more hydrogen with increasing pressure as more C-C and C-N bonds form. This effect has been reported previously (36, 37). We show results from oxygen-free simulations δ and ϵ in this figure to emphasize that this effect occurs under conditions that are found deep in the mantles of Uranus and Neptune (Fig. 5). By comparing the H-H peak in panels (*A* and *B*) of Fig. 4, one finds that more hydrogen atoms are close to each other at higher pressure. To emphasize this result, we determined the nearest neighbor of every hydrogen atom and classified the neighbors by atom type. While for simulation δ at 228 GPa, 14% and 5% of the hydrogen reported carbon and nitrogen atoms as nearest neighbors, those fractions dropped to 7% and 3% in simulation ϵ at 580 GPa. When we conducted the same analysis for the simulations α and β that include oxygen, we find that 25% and 46% of the hydrogen atoms report oxygen as their nearest neighbor. This confirms that there is a strong trend of hydrogen to bond with oxygen.

Finally, we performed conductivity calculations at conditions close to the boundary between the water-rich and C-N-H layers in Fig. 5. In *SI Appendix*, Fig. S1, we show electronic density of state (DOS) to illustrate that all fluids have a metallic character because instead of a gap, they all exhibit a finite DOS value at the Fermi energy. For silicate liquids, this value was reported to be approximately 0.006 (38) while in *SI Appendix*, Fig. S1, we find values between 0.10 and 0.25 depending on composition. For a O_21_N_3_C_12_H_99_ fluid at 4,000 K and 232 or 289 GPa, we calculated a conductivity of 7,000 or 11,000 S/cm, respectively. For fluids of composition O_84_H_228_ and O_84_H_396_ at 244 and 256 GPa, we derived a conductivity of 8,000 or 21,000 S/cm, respectively. This shows that the presence of more hydrogen leads to a higher DOS at the Fermi energy (*SI Appendix*, Fig. S1) and increases the conductivity. The highest values for the DOS and the conductivity were obtained for a C_48_N_12_H_114_ fluid at 200 and 251 GPa, for which we derived a conductivity of 31,000 and 35,000 S/cm, respectively. This means all fluids under consideration are good electrical conductors and may contribute to the generation of magnetic dynamos.

## Predictions from Interior Models

The ab initio simulations showed that a mixture of H_2_O, CH_4_, and NH_3_ will phase separate at high pressure into a water-rich fluid and a C-N-H fluid. With increasing pressure, more and more hydrogen is released from the C-N-H mixture. When we construct models for the interiors of Uranus and Neptune, we introduce a number of additional simplifying assumptions. First, we assume that water-rich fluid separates completely from the C-N-H fluid, each forming a distinct layer in the planetary interior. The hydrogen content of the C-N-H layer decreases with increasing pressure, which makes the hydrogen-poor fluid in the deeper part of this layer more dense than it would be from the pressure-driven compression alone. More importantly, this gradient in hydrogen contents makes this layer stable against convection. While fluids may still move horizontally, vertical motion is prevented by the gradients in composition and density, which implies this layer cannot contribute significantly to the magnetic field generation. We propose this primarily occurs in the upper, water-rich layer that we assume to be homogeneous and convective. For this layer to be less dense than the C-N-H fluid below, some of the hydrogen, which was released from the C-N-H layer, was absorbed into the water-rich layer above. Experiments (39) and ab initio simulation (40) have shown water and hydrogen to be soluble at high pressure. Water (see citation in ref. 12) and hydrogen (33) have also been shown to become electrical conductors at high pressure (Fig. 1). Our interior models for Uranus and Neptune thus have four layers: an outer hydrogen–helium layer, followed by a water-rich layer where the magnetic field is primarily generated, a C-N-H layer with varying hydrogen contents and a rocky core as illustrated in Fig. 5.

We introduce the parameters $H_{2}$ and $H_{3}$ that respectively define the hydrogen fractions at the top and the bottom of the C-N-H layer that spans the region between the equatorial radii $r_{2}$ and $r_{3}$. Both H values are defined in terms of the number of carbon and nitrogen atoms in the fluid, $N_{C}$ and $N_{N}$,

[2] $$\begin{array}{c} H_{2,3}=\frac{N_{H}}{4N_{C}+3N_{N}}\;, \end{array}$$

so that $H_{2,3}$ equals 1 if no hydrogen was released from the CH_4_–NH_3_ mixture. For stable stratification, we require $H_{3}<H_{2}$. We derive the hydrogen contents through the C-N-H layer by linearly interpolating between these two values as a function of equatorial radius, $H\left(r\right)=H_{3}+\left(H_{2}-H_{3}\right)\times \left(r-r_{3}\right)/\left(r_{2}-r_{3}\right)$. For the hydrogen contents of the water-rich layer, we introduce the parameter, $H_{1}$, that we define in terms of the number of oxygen atoms, $N_{O}$,

[3] $$\begin{array}{c} H_{1}=\frac{N_{H}}{2N_{O}}\;, \end{array}$$

so that $H_{1}$ equals 1 and 2 corresponds to the compositions H_2_O and H_4_O, respectively.

To construct interior models, we also need to derive the density as a function of pressure, temperature, and hydrogen fraction for the water-rich and C-N-H layers. So over the pressure–temperature range in Fig. 1, we performed ab initio simulations of O_84_H_226_, O_84_H_282_, and O_84_H_396_ for $H_{1}=2.69,3.36$, and 4.71. To characterize the density profile in the C-N-H layer we conducted simulations of C_48_N_12_, C_48_N_12_H_58_, C_48_N_12_H_114_, and C_48_N_12_H_228_ for $H_{2,3}=0,0.254,0.5$ and 1 over a similar pressure–temperature range. The density is then derived via interpolation as a function of pressure, temperature, and hydrogen fraction. We do not compute adiabats here but instead adopted the pressure–temperature profiles that Redmer et al. (32) derived for Uranus and Neptune. For the core, we assume iron–silicate mixture with a terrestrial iron fraction of 0.325. The equations of state are taken from refs. 41 and 42. We assume the outermost layer is composed of hydrogen and helium in protosolar proportions, $m_{\mathrm{He}}/\left(m_{H}+m_{\mathrm{He}}\right)=0.274$ (34). The equations of state from refs. 43 and 44 were employed.

**Fig. 6.**
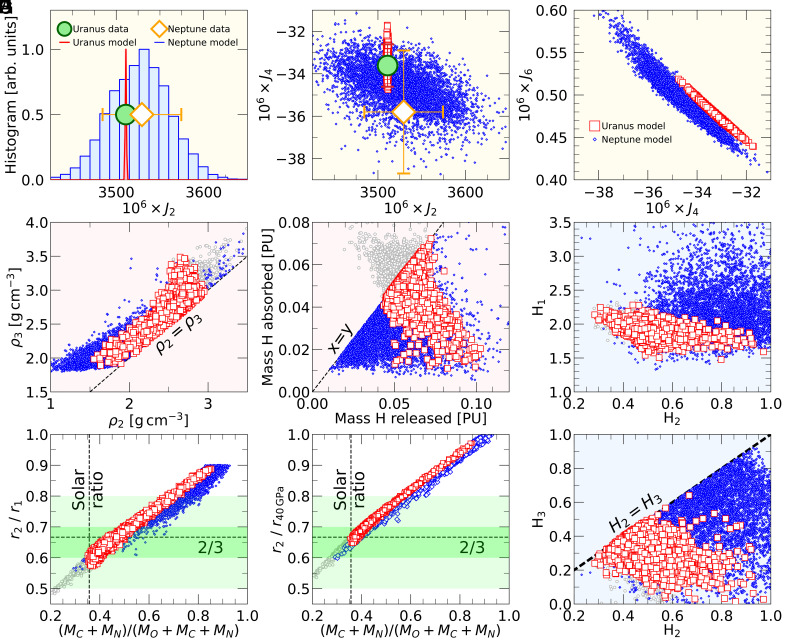
Results from ensembles of interior models for Uranus and Neptune. Panels (*A*–*C*) display the computed gravity coefficients and compare them with measured values of $J_{2}$ and $J_{4}$. Panel (*D*) compares the density at the *Lower* end of the water-rich layer, $\rho_{2}$, with that at the *Upper* end of the C-H-N layer, $\rho_{3}$. Unstable models with $\rho_{2}>\rho_{3}$ were excluded. Panel (*E*) compares the mass of hydrogen in planetary units that was released from the C-N-H layer, x, with the mass of hydrogen that was absorbed by the water-rich layer, y. Models with y>x are disfavored but we show such models for Uranus in gray color. Panel (*F*) shows the radius ratio of the *Lower* and *Upper* radii of the water-rich layer, $r_{2}/r_{1}$, and compared it to the fiducial value of 2/3 that was proposed by Stanley and Bloxham (11). The green bands show plausible radius ratios of 0.6 to 0.7 and 0.5 to 0.8 from Stanley and Bloxham (12). On the horizontal axis, we plot the mass of the carbon and nitrogen nuclei in the planet divided by the combined mass of carbon, nitrogen, and oxygen. We compare it with the plausible, proto-solar value of 0.357 (34). In panel (*G*), we plot the ratio of $r_{2}$ and the radius at 40 GPa that was assumed to be the outer pressure of the dynamo active layer in Ref. (12). Panels (*H*) and (*I*) compare $H_{1}$, $H_{2}$, and $H_{3}$ that specify the hydrogen fractions in the water-rich and C-N-H layers. Unstable models with $H_{3}>H_{2}$ were excluded.

We construct ensembles of interior models with four layers using the nonperturbative Concentric MacLaurin Spheroid (CMS) method (see *Materials and Methods* section for details). We followed the work by Nettelmann et al. (23) when we made the following, plausible assumptions. For Uranus and Neptune, we adopted the equatorial radii of 25,559 and 24,766 km, planet masses of 14.536 and 17.148 Earth masses, rotation periods of 17:14:40 and 16:06:40 h, and 1 bar temperatures of 76 and 72 K. The mass and equatorial radius also define a set of planetary units (PU) for each planet. The target values for gravitational moments were $J_{2}\times 10^{6}=3,510.99\pm 0.72$ and 3,529±45 as well as $J_{4}\times 10^{6}=-33.61\pm 1$ and −35.8±2.9 as determined by the Voyager 2 spacecraft. For every model, we compute the $\chi^{2}$ deviation between the measured and calculated values and then employed our quadratic Monte Carlo method (45) to construct the ensembles of models for both planets that we show in Fig. 6.

Under these assumptions, we have no difficulties matching the measured gravity coefficients $J_{2}$ and $J_{4}$ within their uncertainties with ensembles of models for Uranus and Neptune as panels (*A* and *B*) of Fig. 6 illustrate. Panel (*C*) makes predictions for the expected range of $J_{6}$, which will eventually be measured with high precision by orbiting spacecrafts. This panel also shows the typical negative correlation between $J_{4}$ and $J_{6}$ that has already been seen for Jupiter (1) even though Jupiter’s gravity coefficients are much larger because this planet rotates more rapidly. Movshovitz and Fortney (7) did not see a strong correlation between $J_{4}$ and $J_{6}$ among their interior models, presumably because a much wide range of agnostic pressure-density profiles was considered. Panels (*H* and *I*) show that $H_{2}$, the hydrogen fraction at the upper end of the C-N-H layer, varies between 0.3 and 0.9 (composition C_8_N_2_H_11_… C_8_N_2_H_34_) for Uranus and between 0.35 and 1.0 (C_8_N_2_H_13_… C_8_N_2_H_38_) for Neptune. The hydrogen fraction at the lower layer boundary, $H_{3}$, varies substantially between zero (no hydrogen) and the imposed upper limit equal to $H_{2}$.

In panel Fig. 6*D*, we compare the density of the lower end of the water-rich layer, $\rho_{2}$, with that of the upper end of the C-N-H layer, $\rho_{3}$. For the majority of our models, we find $\rho_{2}<\rho_{3}$, which renders the interface between the two layers stable. For a few models, this stability condition is not satisfied and have thus eliminated them from consideration.

Panel Fig. 6*E* shows that in all of our models, some hydrogen has been absorbed by the water-rich layer, at least 1% of the planet’s total mass. Values up to 7% are permitted. There are many models where all the released hydrogen was incorporated into the water-rich later (x=y). But there are also models where 0.1 PU worth of hydrogen were released but only 0.01 PU was absorbed by the water-rich layer above.

For the ratio, ξ, between the lower and upper radii of the dynamo active layer, Stanley and Bloxham (11) suggested the fiducial value of 2/3. In ref. 12, they obtained reasonable magnetic fields for ξ values between 0.5 and 0.8 and the best results for 0.6 to 0.7. Panels (*F* and *G*) of Fig. 6 show that we can reproduce these values very well but that requires some discussion. In panel (*F*), we plot the ratio of the lower and the upper radii of the water-rich layer in our models, $r_{2}/r_{1}$. In panel (*G*), we divide by the radius of 40 GPa level because Stanley and Bloxham (12) chose this pressure for the upper boundary of their dynamo active layer because experiments have shown that H_2_O attains a nominal electrical conductivity of 20 S/cm. They associated 40 GPa with a fractional radius of 0.7 in Uranus and 0.8 in Neptune. Our detailed interior models place the 40 GPa level at 0.730 and 0.788 respectively. Ref. 21 pointed out that the upper end of the dynamo region is uncertain.

For our Uranus models in Fig. 6*G*, the ratio of $r_{2}/r_{40\;\mathrm{GPa}}$ varies between 0.64 and 0.98. Radius ratios as low as 0.53 become possible if we assume some additional hydrogen was incorporated into the water-rich layer during formation and thus lift the condition that the water-rich layer cannot contain more hydrogen than the C-N-H layer has released (gray symbols). The radius ratio tightly correlates with oxygen to carbon+nitrogen mass ratio, which is expected because the rocky cores are not very massive.

There are many Uranus and Neptune models in the preferred $r_{2}/r_{40\;\mathrm{GPa}}$ range from 0.6 to 0.7, which also have a mass ratio that is close to the protosolar of $\eta =\left(M_{C}+M_{N}\right)/\left(M_{O}+M_{C}+M_{N}\right)=0.357$. We thus conclude that our assumption that the planetary ices were delivered to Uranus and Neptune in protosolar proportions is broadly compatible with the layer thicknesses that Stanley and Bloxham inferred from the observed magnetic field morphology.

In Fig. 5 and Table 1, we compare two representative models for Uranus and Neptune that were selected because their mass ratio, η, was close but still slightly above the protosolar value of 0.357. The measured gravity coefficients, $J_{2}$ and $J_{4}$, are reproduced very well. Since Neptune is more massive, the density throughout its interior is higher. Its core mass and central pressure are higher as well but its outer hydrogen–helium layer is thinner. In both models, the water-rich layer comprises approximately 60% of the planet’s mass while the C-N-H layer contains about 1/3. Almost all hydrogen that was released from the C-N-H layer was absorbed into the water-rich layer leading to a composition of approximately H_4_O. The radius ratio $r_{2}/r_{40\;\mathrm{GPa}}$ are ∼0.66 so within the proposed 0.6 to 0.7 range (12) and close the canonical value of 2/3 (11).

**Table 1.** Parameters of Uranus and Neptune models in Fig. 5

	Uranus	Neptune
Measured *J*_2_ × 10^6^	3,510.99 ± 0.72	3,529 ± 45
Model *J*_2_ × 10^6^	3,510.99	3,529.40
Measured J4 × 10^6^	−33.61 ± 1	−35.8 ± 2.9
Model J4 × 10^6^	−33.61	−35.80
Model J6 × 10^6^	0.4859	0.5314
*H*_1_	1.923 ≈ H_3.8_O	2.245 ≈ H_4.5_O
*H*2	0.5015 ≈ C_8_N_2_H_19_	0.4418 ≈ C_8_N_2_H_17_
*H*3	0.2053 ≈ C_8_N_2_H_8_	0.1055 ≈ C_8_N_2_H_4_
*r*_1_ [PU]	0.8156	0.8858
*r*_2_ [PU]	0.4897	0.5232
*r*_3_ [PU]	0.1471	0.2159
*r*_2_/*r*_1_ (volumetric radii)	0.6010	0.5915
*r*_2_/*r*_40_ (volumetric radii) GPa	0.6680	0.6613
$\frac{M_{C}+M_{N}}{M_{O}+M_{C}+M_{N}}$	0.373	0.400
*M*_H absorbed_ [PU]	0.05606	0.06902
*M*_H released_ [PU]	0.05607	0.06906

## Conclusions

With ab initio simulations, we predict a homogeneous mixture of planetary ices to phase separate at high pressure into a water-rich and a C-N-H dominated fluid. Both are predicted to be good electrical conductors. The phase separation leads to a signature in the structure factor that can be probed with X-ray diffraction experiments. We have thereby derived a testable, material-based explanation for why Uranus and Neptune have nondipolar magnetic fields. Our work supports the hypothesis by Stanley and Bloxham (11, 12) who predicted that the field is generated only in a thin outer layer. We predict it to be a mixture of water and hydrogen. We further predict the lower layer to be a C-N-H dominated fluid that is stably stratified because its hydrogen content decreases with depth. Such a stratification modifies the normal mode spectrum of ice giant planets, which provides strong motivation to bring a Doppler imager on a future mission to Uranus. An entry probe could measure the hydrogen–helium ratio. If this value were found to be larger than protosolar, it would imply that the water-rich layer did not absorb all hydrogen that was released by the C-N-H layer below.

## Materials and Methods

### Ab Initio Simulations.

All density functional molecular dynamics (MD) calculations were performed with version 6 of the Vienna Ab Initio Package (VASP). We employed the Perdew, Burke, and Ernzerhof functional (46) and used hard pseudopotentials with the projector augmented-wave method (47). The valence configurations for the atoms were O([He]2s^266^2p^267^), N([He]2s^268^2p^269^), C([He]2s^270^2p^271^), and H(1s^272^). The initial set of simulations were performed with 12 × [7H_2_O + 4CH_4_+ NH_3_] = O_84_C_48_N_12_H_396_ nuclei and for a system with a hydrogen reduced concentration, O_84_N_12_C_48_H_232_. To study the water-rich layer, we performed simulations with O_84_H_226_, O_84_H_282_, and O_84_H_396_ nuclei and combined with results from ref. 30. For the C-N-H layer, we conducted simulations with C_48_N_12_, C_48_N_12_H_58_, C_48_N_12_H_114_, and C_48_N_12_H_228_ atoms. All equation of state calculations employed the on-the-fly machine learning (ML) method to accelerate the original Kohn–Sham MD method but still employ it at selected steps. At no point did we rely on a fitted ML force field alone. Furthermore the simulations α−δ in Fig. 2 employed only the original Kohn–Sham MD method to ensure that the predicted phase separation is not the result of any ML approximations. All molecular dynamics simulations used a 0.2 fs timestep. The temperature of our NVT ensembles was regulated by a Nosé-Hoover thermostat (48). For the MD simulations, the electronic wave functions were expanded in a plane-wave basis with an energy cutoff of 700 eV and we used the Γ-point to sample the Brillouin zone of our supercells. We followed ref. 38 when we computed the electrical conductivity for different compositions using the Kubo–Greenwood formalism as implemented in the VASP code. For ten equally spaced snapshots on the existing ML-free trajectories, we averaged the conductivity that we calculated with the Heyd, Scuseria, and Ernzerhof functional (49) while employing a 2 × 2 × 2 k-point grid, a 1,100 eV energy cutoff, and additional bands. We estimate the uncertainty of the predicted conductivity values to be 30%, which mainly arises from the required extrapolation to the zero-frequency limit.

### Planetary Interior Models.

All interior models were constructed with the nonperturbative CMS method (27, 28), which decomposes the interior of a rotating planet into a series of $N_{S}=512$ spheroids and then adjusts their shapes until a state of hydrostatic equilibrium is established. While the rotation period and the equatorial radius are matched by construction, reproducing the planet’s total mass requires some care. In ref. 50, the density of the innermost spheroid was adjusted to match Jupiter’s total mass. In refs. 1 and 51, we varied the heavy element abundances of the outer and inner layers to match Jupiter’s mass and $J_{2}$. To match the planet’s total mass here, we step-by-step scale the equatorial radii of all three inner layers, $r_{1}$, $r_{2}$, and $r_{3}$ as the CMS method converges to a hydrostatic solution. To reduce the discretization error, we also slightly adjust the λ grid of the equatorial spheroid radii so that every layer boundary coincides with a grid point. A comparison we conducted calculations with $N_{S}=256$ spheroids and found that the change in the predicted gravity coefficients is small. [Bailey and Stevenson (25) used $N_{S}=30$ spheroids for their Uranus and Neptune models.] Scaling the radii $r_{1}$, $r_{2}$, and $r_{3}$ enables us to derive a valid interior model of the expected mass even in situations where one of these three layers is rather small. This also means that we have removed one dimension from our Monte Carlo calculations and now have five independent parameters, $r_{2}/r_{1}$, $r_{3}/r_{1}$, $H_{1}$, $H_{2}$, and $H_{3}$ that are constrained to satisfy $1\leq r_{2}/r_{1}\leq r_{3}/r_{1}\leq 0$ and $H_{2}>H_{3}$.

## Supplementary Material

Dataset S01 (TXT)

Dataset S02 (TXT)

Dataset S03 (TXT)

Dataset S04 (TXT)

Dataset S05 (TXT)

Dataset S06 (TXT)

Dataset S07 (TXT)

## Data Availability

Data files for all figures, our interior models for Uranus and Neptune in Fig. 5, and equations of state in Fig. 1 are available online (52, 53). Our CMS code is available online (54).
